# Composition and function of rhizosphere microbiome of *Panax notoginseng* with discrepant yields

**DOI:** 10.1186/s13020-020-00364-4

**Published:** 2020-08-10

**Authors:** Mengzhi Li, Zhongjian Chen, Jun Qian, Fugang Wei, Guozhuang Zhang, Yong Wang, Guangfei Wei, Zhigang Hu, Linlin Dong, Shilin Chen

**Affiliations:** 1 College of Pharmacy, Hubei University of Chinese Medicine, Wuhan, 430065 China; 2 Key Laboratory of Beijing for Identification and Safety Evaluation of Chinese Medicine, China Institute of Chinese Materia Medica, Academy of Chinese Medical Sciences, Beijing, 100700 China; 3 Institute of Sanqi Research, Wenshan University, Wenshan, 663000 China; 4 Wenshan Miaoxiang Notoginseng Technology, Co., Ltd, Wenshan, 663000 China; 5 College of Pharmaceutical Science, Dali University, Dali, 671000 China

**Keywords:** Rhizosphere microbiome, *Panax notoginseng*, Metagenome, Yields, Continuous cropping, Functional traits

## Abstract

**Background:**

*Panax notoginseng* is a highly valuable medicinal plant. Reduced *P. notoginseng* yield is a common and serious problem that arises in a continuous cropping system. Variation in the composition and function of soil microbial community is considered the primary cause of yield reduction.

**Methods:**

This study used shotgun metagenomic sequencing approaches to describe the taxonomic and functional features of *P. notoginseng* rhizosphere microbiome and screen microbial taxa and functional traits related to yields.

**Results:**

At the family and genus level, a total of 43 families and 45 genera (relative abundance > 0.1%) were obtained, and the correlation with the yield of *P. notoginseng* was further analyzed. Nitrosomonadaceae, Xanthomonadaceae, *Mycobacterium* and *Arthrobacter* that were enriched in soils with higher yields were positively correlated with *P. notoginseng* yields, thereby suggesting that they might increase yields. Negative correlation coefficients indicated that Xanthobacteraceae, Caulobacteraceae, Oxalobacteraceae, Chitinophagaceae, *Sphingomonas*, *Hyphomicrobium*, *Variovora*x and *Phenylobacterium* might be detrimental to *P. notoginseng* growth. A total of 85 functional traits were significantly (*P* < 0.05) correlated with *P. notoginseng* yields. Functional traits, likely steroid biosynthesis and MAPK signaling pathway were positively correlated with *P. notoginseng* yields. In contrast, functional traits, such as bacterial secretion system, ABC transporters, metabolism of xenobiotics by cytochrome P450 and drug metabolism–cytochrome P450, were negatively associated with yields.

**Conclusions:**

This study describes an overview of the rhizosphere microbiome of *P. notoginseng* with discrepant yields and identifies the taxa and functional traits related to yields. Our results provide valuable information to guide the isolation and culture of potentially beneficial microorganisms and to utilize the power of the microbiome to increase plant yields in a continuous cropping system.

## Background

Sanchi ginseng [*Panax notoginseng* (Burk.) F. H. Chen] belongs to the Araliaceae family and is well known for its therapeutic effects [1–3], such as antihypertensive, antithrombotic, anti-atherosclerotic, anti-tumour, anti-oxidant and hepatoprotective activities [4–7]. *P. notoginseng* is used to treat cardiovascular diseases, inflammation, various body pains, trauma and internal and external bleeding due to injury [8]. At present, *P. notoginseng* roots are used as raw materials in more than 400 Chinese medicinal products of 1300 companies in China [9]. This species is in high demand annually in global market as a valuable traditional medicine. However, wild *P. notoginseng* resources have dwindled, and *P. notoginseng* is cultivated to satisfy market demands. *P. notoginseng* is a perennial medicinal plant cultivated in fixed plots for at least 3 years to obtain high quality roots [10, 11], and continuous cropping can decrease tuber quality and yield [12]. *P. notoginseng* has a narrow ecological range, and its cultivation primarily occurs in Wenshan, Yunnan Province [11, 13]. Approximately 8–10 years of crop rotation is needed to replant *P. notoginseng* [11, 14]. Nowadays, arable soils for *P. notoginseng* cultivation are becoming scarce [11]. The continuous cropping obstacle is a major constraint that hinders the sustainable development of *P. notoginseng* industry, thereby requiring urgent resolution.

Various factors have been considered to contribute to continuous cropping obstacle, including deterioration of soil physicochemical properties, soil-borne diseases, nutrient imbalance, changes in soil microbial communities and accumulation of autotoxic substances [15–18]. Soil microorganisms play an important role in soil function, especially in regulating plant growth, yield and quality [19, 20]. The diversity and composition of soil microbial communities are critical to maintain soil health [21, 22]. Many previous findings have demonstrated that continuous cropping is commonly linked to the disruption of the soil microbial community and regarded as a cause of reduced yield in *Pisum sativum* L., *Rehmannia glutinosa*, *Arachis hypogaea* L. and *Malus pumila* [16, 23–25]. Changes in soil microbial diversity and composition from continuous cropping system negatively affect soil productivity and crop yield [25, 26]. Continuous monoculture of peanut increased pathogenic fungal and simplified beneficial fungal community, thereby contributing to the decline in peanut growth and yield [23]. The balance of soil microbial communities was disrupted in the continuous cropping practices of *P. notoginseng*. Moreover, the bacterial number and diversity in rhizosphere soil of *P. notoginseng* decreased with the increasing duration of continuous cropping [9]. Previous studies have focused on the changes of rhizosphere microorganisms in *P. notoginseng* in a continuous cropping system. However, information about soil microbial taxa related to *P. notoginseng* yield is rare. Identification of beneficial microbial communities that are relevant to plant yield contributes to the exploitation of the functional microbe to promote soil microecological environment in a continuous cropping system.

Amplicon sequencing approach is a classical method used to analyse microbial diversity and composition [27], thereby revealing the variations of microbial community in soils that underwent continuous cropping [14, 24–26]. Metagenomic shotgun sequencing can also determine the identity and relative abundance of microbes that are undetectable using amplicon sequencing approaches due to primer bias [27, 28]. Metagenomic analysis can provide taxonomic, genomic and functional information of the entire community of microorganisms at a given site [27, 29]. However, the relatively high costs of shotgun metagenomics and more demanding bioinformatic requirements have precluded its use for microbiome analysis on a wide scale [30, 31]. Many functional traits primarily involved in plant–microbe and microbe–microbe interactions are closely related to plant health and growth, and have been detected using metagenomic sequencing analysis [32]. Metagenomic sequencing analysis effectively reveals the microbial function of soil microbiome to reflect the ecological process in agricultural practice. Currently, the profiles of rhizosphere microbiome in the continuous cropping system using metagenomic sequencing analysis is rarely reported. A comprehensive understanding of rhizosphere microbial composition and function of *P. notoginseng* with discrepant yields will have significant agricultural implications, including maximizing the use of beneficial microbes and microbial derivatives to increase the yield of medicinal plants.

In this study, the structure and function of rhizosphere microbiome in the soils of *P. notoginseng* plants with different yields were investigated using metagenomic sequencing. We specifically aimed to do the following: (i) clarify the diversity, composition and function of soil microbiome in *P. notoginseng* plants; and (ii) elaborate the relationships among the microbial community and functional traits and *P. notoginseng* yield. Our study presented a comprehensive taxonomic and functional analysis of *P. notoginseng* rhizosphere microbiome by performing metagenomic sequencing, thereby establishing a foundation for the harnessing of the microbiome to improve *P. notoginseng* yield in a continuous cropping system.

## Materials and methods

### Experimental design and sample collection

Roots and rhizosphere soils were collected at *P. notoginseng* harvest stages (3-year-old root growth stages) to explore the relationship between rhizosphere microbiome and yields. *P. notoginseng* roots and rhizosphere soils were collected from Pingba (23°14′29.5″N, 104°5′3.0″E, 1767 m a.s.l.), Yanshan (23°34′56.22″N, 104°19′49.05″E, 1554 m a.s.l.) and Qiubei (23°49′46.99″N, 104°06′12.99″E, 1631 m a.s.l.), Yunnan Province, China, where are the main production regions of *P. notoginseng*. Three experimental sites in the region of Pingba, namely, Pingba A, Pingba B and Pingba C, were used. *P. notoginseng* was cultivated in strict accordance with the standard operating procedures established by the Good Agriculture Practices [33, 34]. The 1-year-old *P*. *notoginseng* seedlings were transplanted in a plantation and cultivated for 2 years before harvest. The experiment was conducted as follows: block design with three replicates in each site; the area of each replicated plot was 1.4 m × 10 m under the same management [2]. Seedlings were removed from 2 m^2^ of each plot, and fresh root weights were analysed to evaluate the *P. notoginseng* yield [35]. The yield was calculated for each plot as the number of weight (kg) divided by the area of each plot (m^2^). Data represent the mean of triplicates. In brief, *P. notoginseng* plants were gently removed from the soils, and rhizospheres were collected by gently shaking the roots to dislodge small adhering soil clumps [36]. Rhizosphere samples were randomly collected from healthy *P. notoginseng* roots (10 plants) in each plot and mixed to form a composite sample. Three replicates were utilised in one site. In total, 15 soil samples were collected, passed through a sieve (2.0 mm) and stored at − 80 °C for DNA extraction.

### DNA extraction and metagenome sequencing

Total genomic DNA was extracted from 0.5 g of soil samples using the MoBio Powersoil Kit (MoBio Laboratories Inc., Carlsbad, CA, USA) according to the manufacturer’s instructions. The DNA quantity and quality of each sample was determined by using a NanoDrop 2000 spectrophotometer (Thermo Scientific, USA) and electrophoresis (1.0% agarose gel, including a 1 kb plus ladder). The DNA samples were stored at − 80℃ until use. Metagenomic library preparation and sequencing were performed following the manufacturer’s protocol at Biozeron, Shanghai, China. Fifteen rhizosphere soil DNA were selected for metagenomic sequencing to evaluate the microbial community structure and function. Metagenomic libraries were constructed using a TruSeq™ DNA Sample Prep Kit (Illumina, USA) according to the manufacturer’s protocol. The metagenomic DNA was sonicated to the 450 bp size range using a Covaris M220. The metagenomic libraries were sequenced on a HiSeq 2500 sequencer (Illumina, USA), and 150-bp paired-end reads were generated.

### Metagenomic analysis

The raw reads from metagenome sequencing were used to generate clean reads by removing adaptor sequences and trimming and removing low quality reads (reads with an N base threshold of 10 and a minimum quality threshold of 20). The clean reads were further trimmed using Sickle software (https://github.com/najoshi/sickle), and trimmed reads that were shorter than 75 bp were discarded. The trimmed reads were mapped to *P. notoginseng* genome using Bowtie2 software [37] to identify and remove the *P. notoginseng* host-originated reads. The optimised sequence reads were assembled de novo by SOAPdenovo (http://soap.genomics.org.cn/, Version 1.06) based on a de Bruijn graph for obtaining contigs. The metagenes were predicted using MetaProdigal (http://prodigal.ornl.gov/) [38]. The non-redundant gene categories (unigenes) were generated using CD-HIT with an identity and average cut-off of 95% and 90%, respectively [39]. The protein sequences were aligned against the NCBI microbial NR database using DIAMOND software [40] with an *E* value cut-off of 1e-5 to generate the taxonomic information of the unigenes. Then, the taxonomic annotations of the unigenes were assigned using the MEGAN LCA algorithm [41]. The functional annotation was assigned to the unigenes by blasting against the KEGG database using BLASTP (BLAST v. 2.2.28 + , http://blast.ncbi.nlm.nih.gov/Blast.cgi) (*e* value ≤ 1*e*−5).

### Statistical analysis

Statistically significant differences in *P. notoginseng* yields, alpha diversity, phylum, family, genus and functional trait were examined using ANOVA test in SPSS 21.0 software (SPSS Institute, Inc., 2010). Significant differences were considered as *P *< 0.05. Alpha diversity (Chao1 and Shannon index) was calculated using QIIME (http://qiime.org/index.html). The taxonomic and functional dissimilarity analyses in *P. notoginseng* rhizosphere soils with different yields were performed using *R* package “vegan” with the Bray–Curtis metric. Principal coordinates analysis (PCoA) was performed using the *R* package “stats” with the Bray–Curtis metric. Pearsonʼs correlation analysis was performed to correlate the *P. notoginseng* yields with the abundance of the microbial taxa and functional traits using SPSS 21.0 software.

## Results

### *P. notoginseng* yields in different sites

**Fig. 1:**
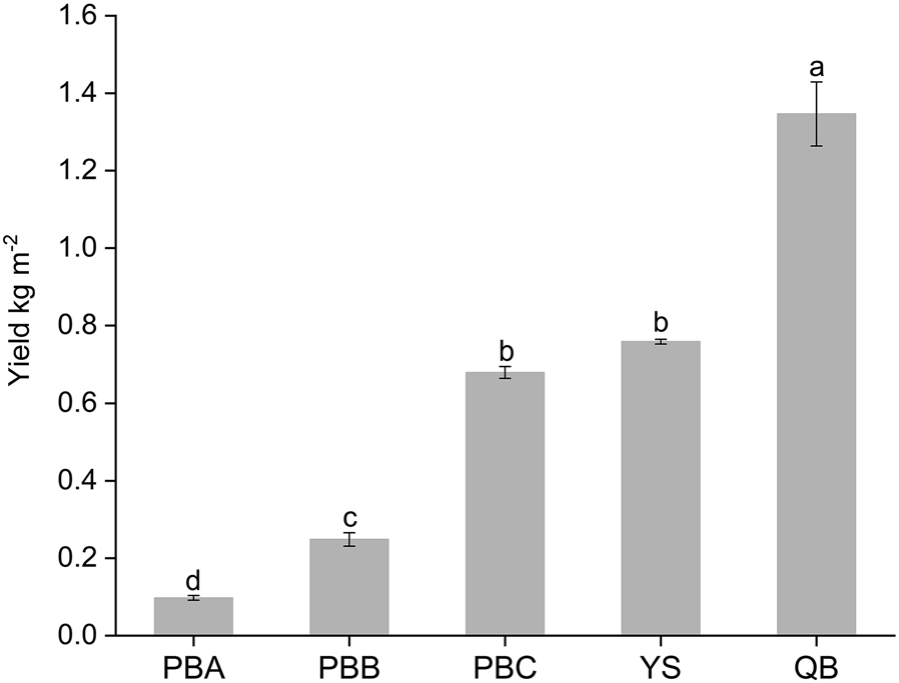
*P. notoginseng* yields. Different letters represent the significant differences between the sample sites at 0.05 significance level. Data represent the mean ± SE (*n *= 3)

*P. notoginseng* yields were significantly diverse in different sites in the range of 0.10–1.35 kg m^−2^ (Fig. 1). *P. notoginseng* yields were 0.10, 0.25, 0.68, 0.76 and 1.35 kg m^−2^ in Pingba A (PBA), Pingba B (PBB), Pingba C (PBC), Yanshan (YS) and Qiubei (QB), respectively. The yield was markedly higher in QB than those of PBA, PBB, PBC and YS (*P *< 0.001).

### Diversity of *P. notoginseng* rhizosphere microbiome

To obtain more information about the composition and function of rhizosphere microbial community of *P. notoginseng* with different yields, rhizosphere DNA was selected for shotgun metagenomic sequencing via a HiSeq 2500 sequencer, after which a total of 1,104,569,780 paired-end clean reads were obtained with approximately 62.9–86.6 million clean reads were obtained per sample (Additional file 1: Table S1). A total of 6,651,849 contigs were generated, with the longest contig at 611,672 bp and N50 at 1083 bp (Additional file 1: Table S2). After removing redundant sequences (identity > 95% and coverage > 90%), 6,228,225 unigenes with an average length of 546.43 bp were generated. The alpha diversity (Chao1 and Shannon index) of the rhizosphere microbiome showed the difference in the sample sites (Table 1). Shannon index at phylum, class, order and family levels were higher in the soils of YS and QB than that in the soils of PBA, PBB and PBC.

**Table 1:** The alpha diversity (Chao1 and Shannon index) of the microbial communities revealed by metagenome data

Sample sites	Phylum	Class	Order	Family	Genus
Chao1					
PBA	174.01 ± 3.45^ab^	291.92 ± 3.76^a^	495.19 ± 2.66^a^	901.77 ± 1.59^a^	2654.37 ± 9.43^a^
PBB	159.75 ± 3.72^b^	283.89 ± 9.67^a^	498.07 ± 12.24^a^	883.88 ± 15.11^a^	2640.47 ± 49.43^a^
PBC	166.37 ± 4.47^ab^	278.42 ± 8.48^a^	485.99 ± 10.88^a^	887.60 ± 28.37^a^	2662.11 ± 54.94^a^
YS	172.20 ± 6.72^ab^	290.10 ± 3.85^a^	506.66 ± 6.37^a^	922.45 ± 16.83^a^	2680.45 ± 18.87^a^
QB	176.32 ± 3.30^a^	293.71 ± 5.05^a^	510.46 ± 17.58^a^	938.83 ± 25.37^a^	2660.01 ± 6.08^a^
Shannon index					
PBA	1.99 ± 0.02^b^	3.40 ± 0.03^b^	4.85 ± 0.03^c^	5.91 ± 0.02^b^	7.27 ± 0.03^a^
PBB	1.99 ± 0.02^b^	3.34 ± 0.07^b^	4.76 ± 0.07^c^	5.86 ± 0.05^b^	7.30 ± 0.04^a^
PBC	2.01 ± 0.12^b^	3.34 ± 0.15^b^	4.77 ± 0.10^c^	5.85 ± 0.09^b^	7.24 ± 0.06^a^
YS	2.63 ± 0.03^a^	3.97 ± 0.04^a^	5.38 ± 0.03^a^	6.25 ± 0.03^a^	7.35 ± 0.03^a^
QB	2.39 ± 0.10^a^	3.70 ± 0.08^a^	5.14 ± 0.09^b^	6.05 ± 0.12^ab^	7.18 ± 0.18^a^

*PBA* Pingba village A, *PBB* Pingba village B, *PBC* Pingba village C, *YS* Yanshan village, *QB* Qiubei village. The mean values of three replicates per site are show, followed by the standard error of the mean. Different letters represent significant difference among five sample sites at the level of 0.05

### Composition of *P. notoginseng* rhizosphere microbiome

**Fig. 2:**
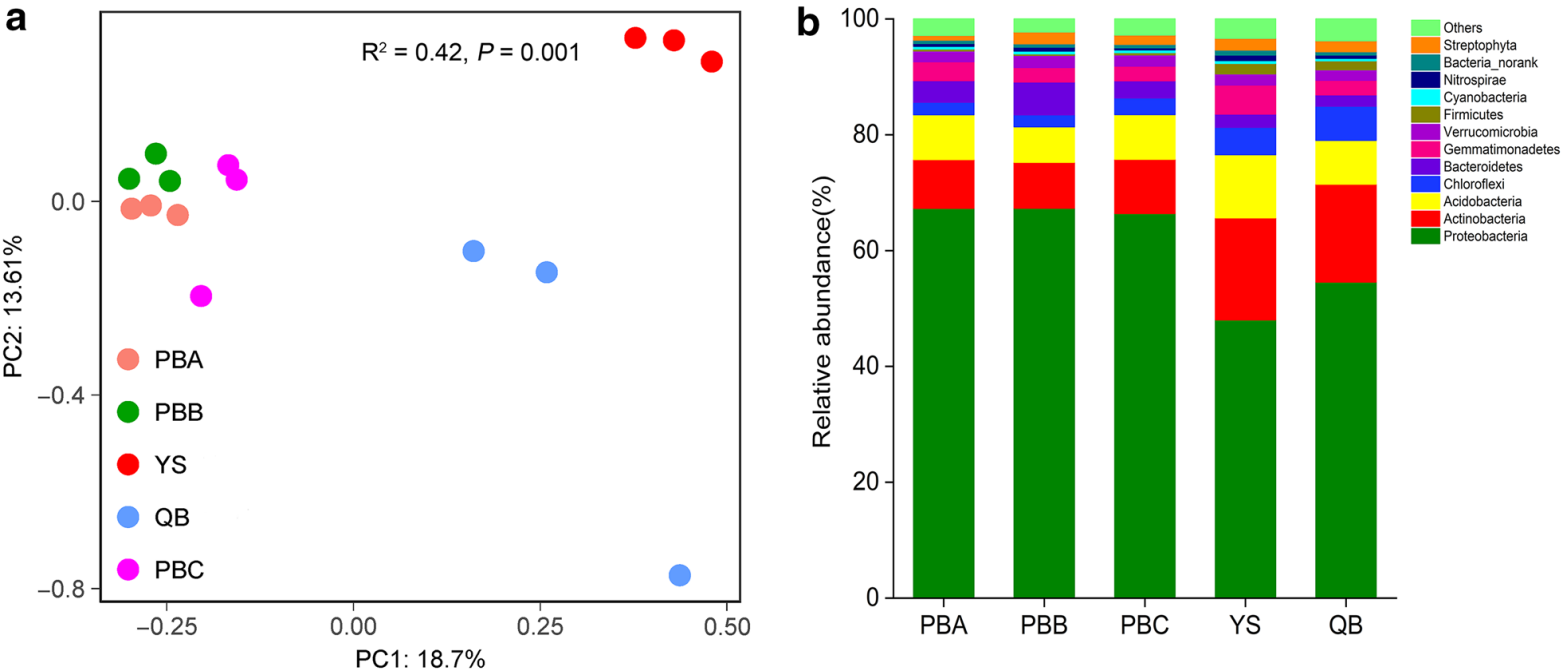
Taxonomic composition of *P. notoginseng* rhizosphere microbiome. **a** PCoA based on the Bray–Curtis distance between different rhizosphere samples. **b** Relative abundance of different phyla in rhizosphere samples

PCoA was performed based on metagenomic sequencing using the Bray–Curtis metric to visualise the difference in microbial communities among soil samples, thereby revealing significant difference in the microbial community in the rhizosphere soils using adonis test analysis (R^2^ = 0.42, *P *= 0.001) (Fig. 2a). The first principal component axis (18.7% contributions) demonstrated that the microbial communities in the soils of YS and QB differed from those of the soils of PBA, PBB and PBC; the second principal component (13.61% contributions) suggested that the microbial communities in the soils of YS significantly differed from those of other sites.

Bacteria is the predominant domain (97.32% ± 0.90%, mean relative abundance ± SD, *n *= 15), with small proportions of Eukaryota, Archaea and Viruses detected based on the annotated unigenes. Proteobacteria, Actinobacteria, Acidobacteria, Chloroflexi, Bacteroidetes, Gemmatimonadetes and Verrucomicrobia were present at high relative abundances (> 1.0%) in the rhizosphere microbiome of sample sites at the phylum level (Fig. 2b). The relative abundance of Proteobacteria was 67.29%, 67.34%, 66.42%, 48.05% and 54.57%, respectively, in rhizosphere soils of PBA, PBB, PBC, YS and QB. The relative abundance of Bacteroidetes was 3.73%, 5.68%, 2.95%, 2.30% and 1.95%, respectively, in rhizosphere soils of PBA, PBB, PBC, YS and QB. The relative abundance of Proteobacteria and Bacteroidetes were significantly higher in rhizosphere soils of PBA, PBB and PBC than YS and QB (*P *< 0.01). The relative abundance of Actinobactera was significantly higher in rhizosphere soils of YS (17.62%) and QB (16.91%) than PBA (8.43%), PBB (7.93%) and PBC (9.36%) (*P *< 0.001).

### Correlations between taxonomic taxa and *P. notoginseng* yields

**Fig. 3:**
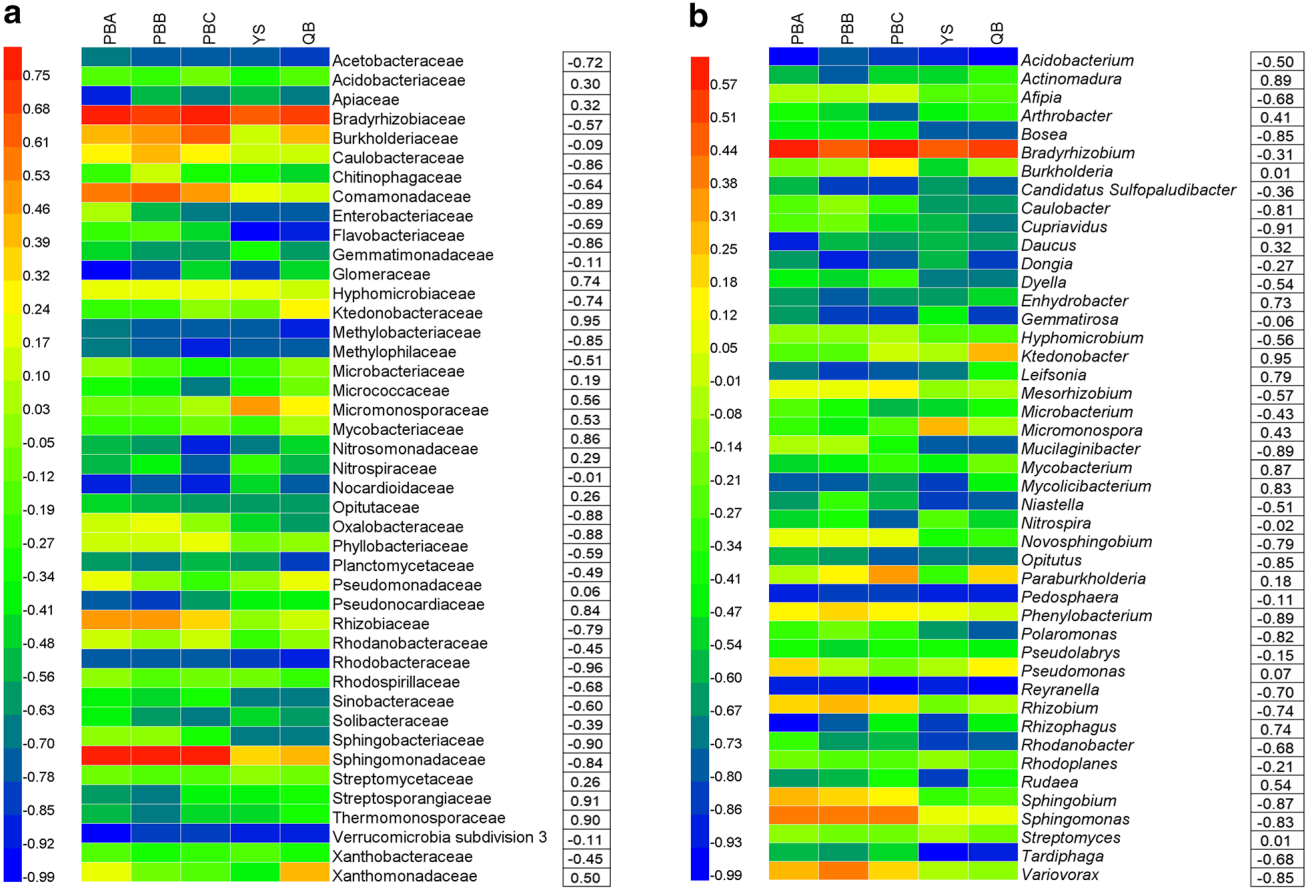
Relative abundance (> 0.1%) of the dominant families **a** and genera **b**, and their Pearson’s correlation coefficients with *P. notoginseng* yields. Data are mean values of *n *= 3

Forty-three families (relative abundance > 0.1%) were obtained from rhizosphere soils of *P. notoginseng* (Fig. 3a). The relative abundance of Caulobacteraceae was 2.08%, 2.45%, 1.94%, 1.42% and 1.27%, respectively, in rhizosphere soils of PBA, PBB, PBC, YS and QB. The relative abundance of Methylobacteriaceae was 0.21%, 0.18%, 0.19%, 0.18% and 0.13%, respectively, in rhizosphere soils of PBA, PBB, PBC, YS and QB. The relative abundance of Caulobacteraceae and Methylobacteriaceae were significantly lower in rhizosphere soils of QB (higher yields) than PBA, PBB, PBC and YS (*P *< 0.05). The relative abundance of Micrococcaceae was significantly higher in rhizosphere soils of QB (0.76%) than PBA (0.49%), PBB (0.40%), PBC (0.21%) and YS (0.50%) (*P *< 0.05). Pearsonʼs correlation analysis showed that the relative abundance of Comamonadaceae (R = − 0.89), Opitutaceae (R = − 0.88), Rhodobacteraceae (R = − 0.96) and Sphingobacteriaceae (R = − 0.90) were negatively related to *P. notoginseng* yield (*P *< 0.05). Relative abundance of Ktedonobacteraceae (R = 0.95), Streptosporangiaceae (R = 0.91) and Thermomonosporaceae (R = 0.90) were positively correlated to *P. notoginseng* yield (*P *< 0.05).

At the genus level, we obtained a total of 45 genera (relative abundance > 0.1%) (Fig. 3b). The relative abundance of *Leifsonia* was significantly higher in rhizosphere soils of QB (0.40%) than PBA (0.21%), PBB (0.15%), PBC (0.17%) and YS (0.20%) (*P *< 0.01). The relative abundance of *Pseudomonas* was significantly higher in rhizosphere soils of PBA (lower yields, 1.55%) than PBB (0.93%), PBC (0.60%), YS (0.90%) and QB (1.48%) (*P *< 0.05). Pearsonʼs correlation analysis showed that the relative abundance of *Actinomadura* (R = 0.89), *Arthrobacter* (R = 0.41), *Enhydrobacter* (R = 0.73), *Leifsonia* (R = 0.79), *Mycolicibacterium* (R = 0.83), *Rhizophagus* (R = 0.74) were positively correlated with *P. notoginseng* yield. Whereas the relative abundance of *Bosea* (R = − 0.85), *Cupriavidus* (R = −0.91), *Mucilaginibacter* (R = − 0.89), *Novosphingobium* (R = − 0.79), *Phenylobacterium* (R = − 0.89), *Opitutus* (R = − 0.85), *Phenylobacterium* (R = − 0.89), *Polaromonas* (R = − 0.82), *Reyranella* (R = − 0.70), *Rhizobium* (R = − 0.74), *Sphingobium* (R = − 0.87), *Sphingomonas* (R = − 0.83) and *Variovorax* (R = − 0.85) were negatively correlated with *P. notoginseng* yield.

### The functional traits of the *P. notoginseng* rhizosphere microbiome

**Fig. 4:**
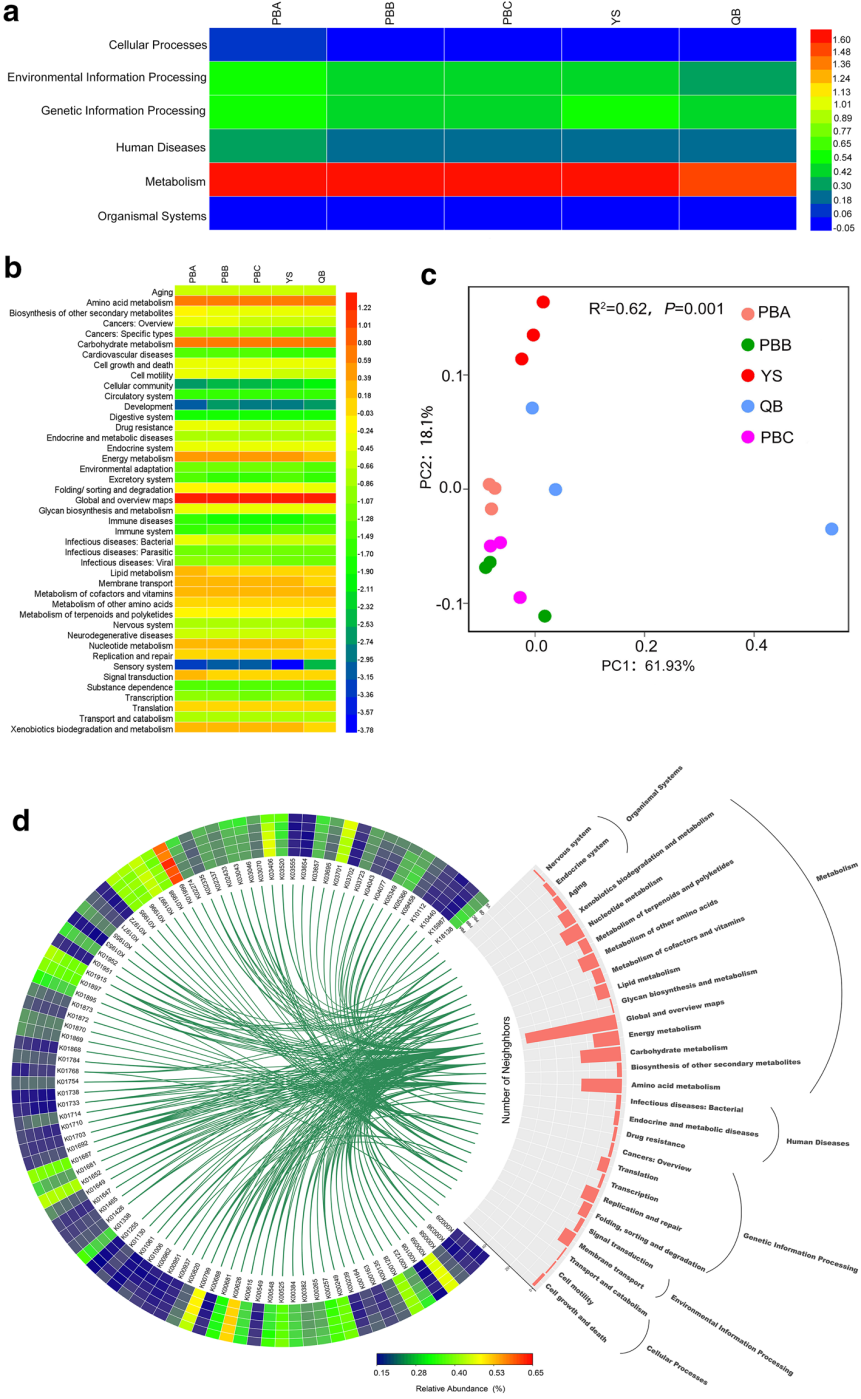
Characterisation of the functional traits of *P*. *notoginseng* rhizosphere microbiome. **a** Functional traits in KEGG level 1 pathway. **b** Functional traits in KEGG level 2 pathway. **c** The principal coordinates analysis (PCoA) of KO functional categories. **d** The relative abindance (≥ 0.15%) of KO functional categories. Data are mean values of *n *= 3

In total, 1,330,812 genes were hit in the KEGG databases and were assigned to 4436 KEGG orthology (KO) functional categories (Additional file 2). The KOs were mainly involved in 6 KEGG level 1 pathways and 43 KEGG level 2 pathways (Fig. 4a, b). The relative abundance of cellular processes, environmental information processing, genetic information processing, human diseases, metabolism and organismal systems pathways were 0.89%–1.24%, 2.56%–3.54%, 2.83%–3.72%, 1.60%–2.08%, 39.62%–51.83% and 0.94%–1.09%, respectively, in rhizosphere soils of five sample sites at the first KEGG level. The relative abundance of cellular processes, environmental information processing, genetic information processing and human diseases were significantly higher in rhizosphere soils of PBA (lower yields) than PBB, PBC, YS and QB (*P *< 0.05). The relative abundance of biosynthesis of other secondary metabolites, drug resistance, environmental adaptation, glycan biosynthesis and metabolism, lipid metabolism, metabolism of cofactors and vitamins, membrane transport and signal transduction were 0.46%–0.61%, 0.28%–0.41%, 0.05%–0.07%, 0.40%–0.55%, 1.24%–1.63%, 1.53%–2.10%, 1.38%–1.98% and 1.18%–1.56%, respectively, in rhizosphere soils of five sample sites at the second KEGG level. The relative abundance of biosynthesis of other secondary metabolites, drug resistance, environmental adaptation, glycan biosynthesis and metabolism, metabolism of cofactors and vitamins, membrane transport and signal transduction were significantly higher in rhizosphere soils of PBA (lower yields) than PBB, PBC,YS and QB (*P* < 0.05). The relative abundance of lipid metabolism was significantly lower in rhizosphere soils of QB (higher yields) than PBA, PBB, PBC and YS (*P* < 0.05).

PCoA was performed based on KO functional categories using the Bray–Curtis metric, and an adonis test (R^2^ = 0.62, *P *= 0.001) showed significant difference in sample sites (Fig. 4c). KO functional categories with a relative abundance exceeding 0.15% are described in Fig. 4d to clarify which KO functional categories were dominant among rhizosphere microbiome of *P. notoginseng* and which metabolism pathways were the main components in sample sites. Ninety-three KO functional categories were obtained with differences among the sample sites. The K01999 (mean 0.61%), K00626 (mean 0.50%), K00799 (mean 0.43%), K00059 (mean 0.42%) and K03701 (mean 0.40%) were the top five categories that were enriched in rhizosphere soils of PBA, PBB, PBC, YS and QB. Notably, the relative abundance of K01999 was the highest among all the KO functional categories, and K00626 was the second highest. K01999, a branched-chain amino acid transport system substrate-binding protein, is a member of membrane transport pathway. K00626 (*atoB*, acetyl-CoA C-acetyltransferase) is involved in carbon metabolism, pyruvate metabolism, carbon fixation pathways in prokaryotes, two-component system, fatty acid metabolism and biosynthesis of antibiotics pathways. K00799 (*gst*, glutathione S-transferase) is involved in glutathione metabolism, metabolism of xenobiotics by cytochrome P450 and drug metabolism–cytochrome P450 pathways. K00059 (*fabG*, 3-oxoacyl-[acyl-carrier protein] reductase) is involved in metabolic, fatty acid metabolism, fatty acid biosynthesis, biosynthesis of unsaturated fatty acids and biotin metabolism pathways. K03701 (*uvrA*, excinuclease ABC subunit A) is mainly involved in the nucleotide excision repair pathway.

### Correlations between functional traits and *P. notoginseng* yields

**Fig. 5:**
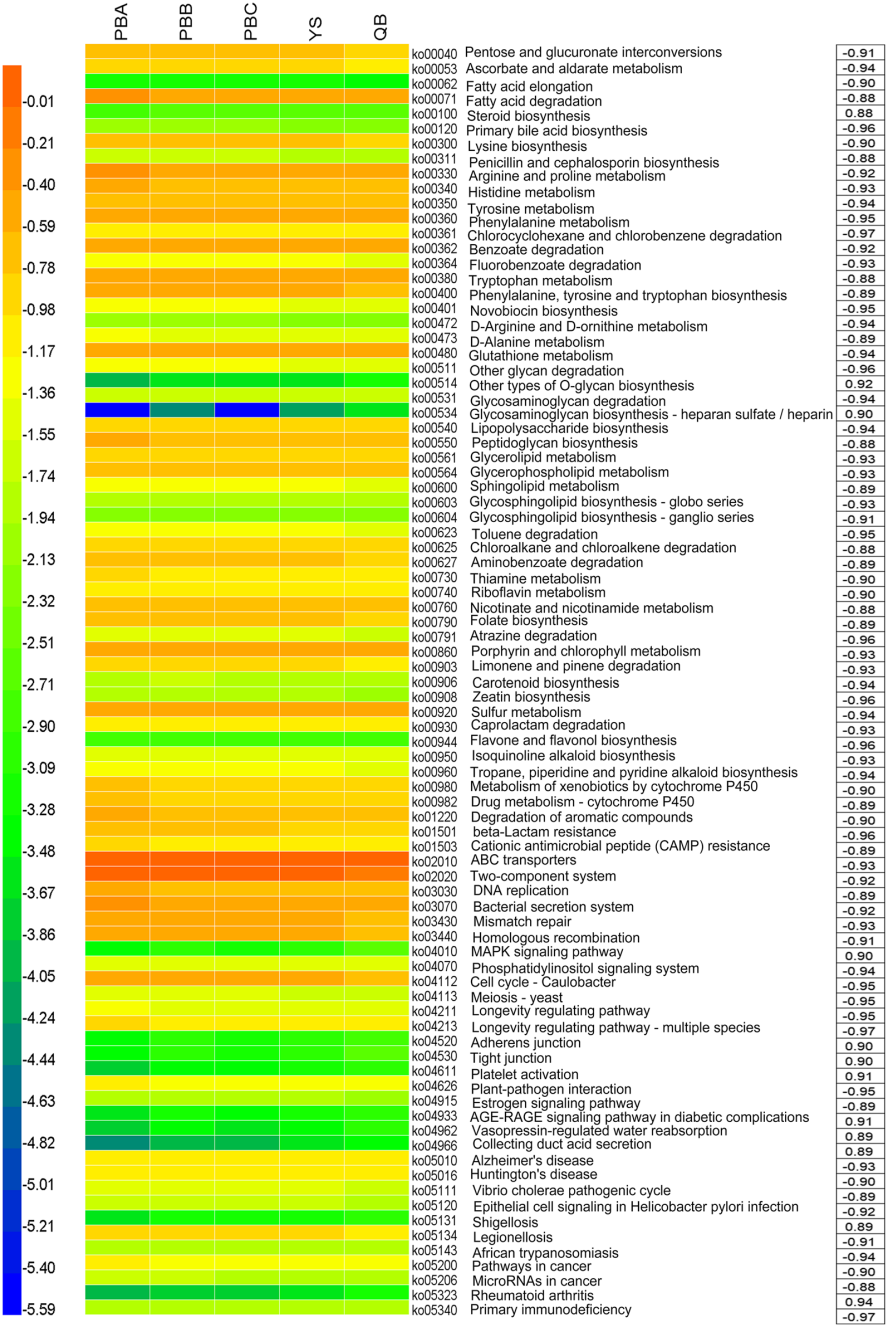
Relative abundance of the functional traits (level 3 of KEGG functional category) and their Pearsonʼs correlation analysis with *P. notoginseng* yields (*P *< 0.05). Date are mean values of *n *= 3

A total of 389 functional classifications were obtained, and the relative abundance showed differences among the sample sites at the third KEGG level (Additional file 3 and Fig. 5). The relative abundance of functional traits, likely steroid hormone biosynthesis (ko00140), lysine biosynthesis (ko00300), ABC transporters (ko02010), two-component system (ko02020) and plant-pathogen interaction (ko04626) were significantly lower in rhizosphere soils of QB (higher yields) than PBA, PBB, PBC and YS (*P *< 0.05). Eighty-five functional traits were significantly correlated (*P *< 0.05) with *P. notoginseng* yields using Pearsonʼs correlation analysis, among 12 and 73 functional traits were positively and negatively correlated with yields, respectively. A total of 304 functional traits were correlated with *P. notoginseng* yields, among which 140 and 164 functional traits were positively and negatively correlated with yields, respectively.

The KOs involved in known plant–microbe and microbe–microbe interactions, such as bacterial secretion system (ko03070), flagellar assembly (ko02040), bacterial chemotaxis (ko02030) and two-component system (ko02020), were negatively correlated with *P. notoginseng* yields (Additional file 3 and Fig. 5). Pearsonʼs correlation analysis showed that the abundances of plant–pathogen interaction (ko04626), ABC transporters (ko02010), metabolism of xenobiotics by cytochrome P450 (ko00980), drug metabolism–cytochrome P450 (ko00982), dioxin degradation (ko00621), chloroalkane and chloroalkene degradation (ko00625) and degradation of aromatic compounds (ko01220) were negatively correlated with *P. notoginseng* yields. Meanwhile the abundances of MAPK signaling pathway (ko04010) and steroid biosynthesis (ko00100) were positively correlated with *P. notoginseng* yields.

## Discussion

This study analysed the taxonomic and functional features of *P*. *notoginseng* rhizosphere microbiome with discrepant yields to determine microbial taxa and functional traits related to yields. Microbial composition exhibited dissimilarity in rhizosphere soils of *P. notoginseng* with discrepant yields, and the findings were consistent with those for other crops [24]. Actinobacteria was more abundant in disease-suppressive soils than in disease-conducive soils in continuous cropping system [42]. And we found that Actinobacteria was enriched in rhizosphere soils of *P. notoginseng* with high yields. Xanthobacteraceae, Caulobacteraceae, Oxalobacteraceae, Phyllobacteriaceae and Chitinophagaceae negatively correlated with *Setaria italica* yields [43]. *Dongia*, O*pitutus*, *Sphingomonas*, *Rhizobium*, *Hyphomicrobium* and *Phenylobacterium* were negatively correlated with apple rootstocks growth [44]. The above taxa were also negatively correlated with *P. notoginseng* yields in our study. *Variovorax* was positively correlated with the *Solanum tuberosum* L. common scab severity level and was supposedly responsible for inducing common scab by stimulating thaxtomin production [45]. Moreover, the high abundances of *Variovorax* were shown in soils with low *P. notoginseng* yields in our study. Overall, *Variovorax* might negatively influence the yields of *P. notoginseng*. Certain microbial communities in the rhizosphere soils could negatively regulate the growth and yields of plants.

Plant growth-promoting rhizobacteria (PGPR) play an important role in enhancing plant health, promoting plant growth and increasing crop yields [46, 47]. Some PGPR, such as Nitrosomonadaceae, Xanthomonadaceae, *Arthrobacter* and *Mycobacterium*, presented positive correlations with *P. notoginseng* yields in our study. Nitrosomonadaceae is used for bioremediation of toxic chemicals in the soil [48]. The addition of Nitrosomonadaceae could reduce nitrogen loss and the time required to stabilise the nitrogen profile [48, 49]. Xanthomonadaceae was positively correlated with foxtail millet yields [43]. *Mycobacterium,* as PGPR genera, had positive effect on plant growth, nutrient uptake and increased root dry weight of maize [46, 47, 50, 51]. *Arthrobacter* acts as antagonistic bacteria against *Sclerotinia sclerotiorum* [52], which can cause *Helianthus annuus* L. sclerotinia rot [53]. The abundance of *Arthrobacter* had significant negative correlation with tobacco bacterial wilt disease [54]. These results indicated that the enrichment of *Mycobacterium* and *Arthrobacter* in soils with higher yields might contribute to the increase of *P. notoginseng* yields. Therefore, regulation of rhizosphere microbiomes contributed to overcome continuous cropping obstacles and increase medicinal plant yields by improving soil environment.

A total of 83 microbial functional traits were significantly correlated (*P *< 0.05) with *P. notoginseng* yields. Moreover, these functional traits contained bacterial secretion system, ABC transporters, metabolism of xenobiotics by cytochrome P450 and drug metabolism–cytochrome P450 which were negatively associated with *P. notoginseng* yields. ABC transporters and bacterial secretion system were enriched in soils of potato with high scab severity level [45], and those functional traits mediated the communication between microorganisms and environments or other organisms [55–57].Thaxtomins are highly phytotoxic cyclic dipeptides produced by plant–pathogenic members of the genus *Streptomyces* [58]. Thaxtomins have the basic structure L-4-nitrotryptophyl-L-phenylalanyl [59], and the biosynthetic pathway of Thaxtomin A (ThxA) involves nitric oxide synthase and cytochrome P450 [60–62]. In this study, the high abundance of xenobiotic metabolism by cytochrome P450, drug metabolism–cytochrome P450 and nitrogen metabolism in low yield soils might be conducive to ThxA biosynthesis. In summary, the occurrence of low *P. notoginseng* yields was accompanied by an increase in the abundance of pathogenicity-related functional traits in rhizosphere microbes. Functional traits involved in MAPK signaling pathway and steroid biosynthesis were positively correlated with *P. notoginseng* yields in the present study. In eukaryotic cells, MAPKs is involved in the transduction of a variety of extracellular signals and the regulation of different developmental processes, such as regulating fungal mating, invasive growth, cell wall integrity and ascospore formation [63, 64]. Steroids can act as signal molecules that mediate communication between microorganisms and hosts [65, 66]. The functional traits involved in MAPK signaling pathway and steroid biosynthesis might benefit *P. notoginseng* yield.

## Conclusion

In summary, the taxonomic and functional properties exhibited dissimilarity in rhizosphere microbiome of *P. notoginseng* with different yields in continuous cropping system based on the shotgun metagenomic sequencing. These microorganisms such as *Arthrobacter* and *Mycobacterium*, and functional traits involved in steroid biosynthesis and MAPK signaling pathway were positively correlated with yields. This work broadens the understanding of the relationship between the rhizosphere microbial composition and function with *P. notoginseng* yields and lays a foundation for the exploitation of microbes to improve soil microecological environment and increase medicinal plant yields in continuous cropping system.

## Supplementary information

**Additional file 1: Table S1.** Summary of the metagenomic reads in five sample sites. **Table S2.** Statistical analysis of rhizosphere soils in *P. notoginseng* from five different sites.

**Additional file 2.** Abundance of the metagenomic microbial function profiling (KEGG orthology function category).

**Additional file 3.** Pearson correlation analysis (*P* ≥ 0.05) among functional traits regarding *P. notoginseng* yields.

## Data Availability

The raw sequencing data are publicly available in the NCBI Sequence Read Archive (SRA) under the Bioproject Number PRJNA595820.

## Abbreviations

PCoA: Principal coordinates analysis
KEGG: Kyoto Encyclopedia of Genes and Genomes
KO: KEGG orthology
PGPR: Plant growth-promoting rhizobacteria
