# Supplementary material for: Composition and function of rhizosphere microbiome of Panax notoginseng with discrepant yields
Source: Chin Med. 2020 Aug 10;15:85. doi: 10.1186/s13020-020-00364-4 (PMC7418314; doi:10.1186/s13020-020-00364-4)
Supplement: Supplementary file 1 — Additional file 1: Table S1. Summary of the metagenomic reads in five sample sites. Table S2. Statistical analysis of rhizosphere soils in P. notoginseng from five different sites. [file 13020_2020_364_MOESM1_ESM.docx]

Supplementary data for

**Composition and function of rhizosphere microbiome of *Panax notoginseng* with discrepant yields**

Mengzhi Li^1,2^, Zhongjian Chen^3,4^, Jun Qian^2,5^, Fugang Wei^4^, Guozhuang Zhang^2^, Yong Wang^3^, Guangfei Wei^2^, Zhigang Hu^1^, Linlin Dong^2*^ and Shilin Chen^1,2*^

*^1^ College of Pharmacy, Hubei University of Chinese Medicine, Wuhan 430065, China*

*^2^ Institute of Chinese Materia Medica, China Academy of Chinese Medical Sciences, Beijing 100700, China*

*^3^ Institute of Sanqi Research, Wenshan University, Wenshan 663000, China*

*^4^ Wenshan Miaoxiang Notoginseng Technology, Co., Ltd., Wenshan 663000, China*

*^5^ College of Pharmaceutical Science, Dali University, Dali, 671000, China*

*Correspondence Author:

Linlin Dong *E-mail*: [lldong@icmm.ac.cn](mailto:lldong@icmm.ac.cn); Shilin Chen *E-mail*: [slchen@icmm.ac.cn](mailto:slchen@icmm.ac.cn);

**This file includes:**

**Table S1.** Summary of the metagenomic reads in five sample sites. **Table S2.** Statistical analysis of rhizosphere soils in *P. notoginseng* from five different sites.

**Other supplementary materials for this manuscript include the following:**

**Additional file 2.** Abundance of the metagenomic microbial function profiling (KEGG orthology function category).

**Additional file 3.** Pearson correlation analysis (*P* ≥ 0.05) among functional traits regarding *P. notoginseng* yields.

**Table S1.** Summary of the metagenomic reads in five sample sites. PBA, Pingba village A; PBB, Pingba village B; PBC, Pingba village C;YS, Yanshan village; QB, Qiubei village.

| Samples | Raw reads | Raw bases (bp) | Clean reads | Clean bases (bp) |
| --- | --- | --- | --- | --- |
| PBA-1 | 89,517,620 | 13,427,643,000 | 86,577,932 | 12,946,825,231 |
| PBA-2 | 77,875,396 | 11,681,309,400 | 73,811,186 | 11,021,753,526 |
| PBA-3 | 80,393,022 | 12,058,953,300 | 75,829,718 | 11,323,895,682 |
| PBB-1 | 90,303,672 | 13,545,550,800 | 86,062,502 | 12,854,364,436 |
| PBB-2 | 79,947,620 | 11,992,143,000 | 75,695,434 | 11,302,424,672 |
| PBB-3 | 80,331,068 | 12,049,660,200 | 75,976,986 | 11,341,978,062 |
| PBC-1 | 84,738,020 | 12,710,703,000 | 80,706,408 | 12,054,737,367 |
| PBC-2 | 72,031,846 | 10,804,776,900 | 68,184,400 | 10,180,107,916 |
| PBC-3 | 74,691,706 | 11,203,755,900 | 70,479,554 | 10,517,678,748 |
| YS-1 | 76,544,788 | 11,481,718,200 | 71,752,240 | 10,707,455,495 |
| YS-2 | 69,942,392 | 10,491,358,800 | 66,235,804 | 9,886,201,626 |
| YS-3 | 73,587,876 | 11,038,181,400 | 69,132,238 | 10,313,940,190 |
| QB-1 | 71,266,910 | 10,690,036,500 | 67,503,716 | 10,077,145,763 |
| QB-2 | 77,472,116 | 11,620,817,400 | 73,680,382 | 11,002,317,780 |
| QB-3 | 67,912,930 | 10,186,939,500 | 62,941,280 | 9,341,692,945 |

-1,-2 and -3 presented three replicates.

**Table S2.** Statistical analysis of rhizosphere soils in *P. notoginseng* from five different sites. PBA, Pingba village A; PBB, Pingba village B; PBC, Pingba village C;YS, Yanshan village; QB, Qiubei village.

| Samples | Contigs | Contigs bases (bp) | N50  (bp) | N90  (bp) | Max  (bp) | Min  (bp) |
| --- | --- | --- | --- | --- | --- | --- |
| PBA-1 | 456,184 | 416,785,299 | 857 | 548 | 167,572 | 500 |
| PBA-2 | 47,4018 | 449,667,410 | 907 | 553 | 236,930 | 500 |
| PBA-3 | 476,899 | 441,829,277 | 892 | 550 | 150,813 | 500 |
| PBB-1 | 589,523 | 581,539,952 | 955 | 552 | 181,330 | 500 |
| PBB-2 | 551,252 | 544,294,322 | 963 | 555 | 318,020 | 500 |
| PBB-3 | 478,458 | 500,254,981 | 1043 | 564 | 230,988 | 500 |
| PBC-1 | 611,672 | 650,793,621 | 1083 | 566 | 518,963 | 500 |
| PBC-2 | 384,434 | 409,059,956 | 1070 | 566 | 297,258 | 500 |
| PBC-3 | 438,511 | 407,187,098 | 902 | 554 | 194,710 | 500 |
| QB-1 | 434,682 | 426,691,868 | 969 | 561 | 173,091 | 500 |
| QB-2 | 458,237 | 450,673,468 | 957 | 557 | 196,678 | 500 |
| QB-3 | 333,438 | 290,318,548 | 797 | 541 | 380,927 | 500 |
| YS-1 | 324,965 | 290,123,530 | 824 | 543 | 935,23 | 500 |
| YS-2 | 314,507 | 277,008,804 | 841 | 551 | 67,721 | 500 |
| YS-3 | 325,069 | 295,000,149 | 851 | 551 | 363,049 | 500 |

-1,-2 and -3 presented three replicates.
